# Describing, Evaluating, and Exploring Barriers to Adoption of Virtual Reality: An International Modified Delphi Consensus Study Involving Clinicians, Educators, and Industry Professionals

**DOI:** 10.1089/jmxr.2024.0022

**Published:** 2024-10-21

**Authors:** Jonathan R. Abbas, Eric Gantwerker, Mark Volk, Tony Payton, Brendan A. McGrath, Neil Tolley, Rachel Isba, Iain A. Bruce

**Affiliations:** 1 The University of Manchester, Manchester, United Kingdom.; 2 Human Factors Academy, Manchester University NHS Foundation Trust, Manchester, United Kingdom.; 3 Donald and Barbara Zucker School of Medicine, Hofstra/Northwell, New York, New York, USA.; 4 Department of Paediatric ORL, Boston Children’s Hospital, Boston, Massachusetts, USA.; 5 Sentira, ^XR^ (trading as VREvo Ltd), Manchester, United Kingdom.; 6 Acute Intensive Care Unit, Wythenshawe Hospital, Manchester University NHS Foundation Trust, Manchester, United Kingdom.; 7 Imperial College Healthcare NHS Trust, St Mary’s Hospital, London, United Kingdom.; 8 Alder Hey Children’s Hospital, Liverpool, United Kingdom.; 9 Lancaster Medical School, Lancaster University, Lancaster, United Kingdom.; 10 Department of Paediatric Otolaryngology, Manchester University NHS Foundation Trust, Manchester, United Kingdom.

**Keywords:** Virtual reality, education, digital literacy, medical education

## Abstract

With the rising interest in extended reality technology, the literature base is rapidly growing. With this growth, confusion within the descriptive terminology, and incoherence in the methodological approach to training tool evaluation exists. Additionally, the development expectations of the health care worker, educator, and academic are not clear. This study aimed to work toward consensus agreement around four key areas of interest: (1) descriptive terminology (2) evaluation methodology (3) research outcome measure selection, and (4) barriers and facilitators to adoption of VR into health care education. This study was an international, electronic, Modified Delphi Consensus study, which ran over two rounds. Systematic literature review, ratified by a multidisciplinary study advisory group, and combined with Delphi round, one participant responses generated a total of 133 propositions across all areas of interest. In two rounds of a Delphi process, consensus was reached for a total of 102 propositions of which 49 were included, and 53 excluded, with the remaining 31 propositions not reaching consensus. Fifteen terms were deemed important when describing VR technology with ‘virtual environment’, ‘interactive’, and ‘simulation’ achieving the highest levels of agreement. There was almost unanimous agreement that technology should be described by its interplay between the hardware and software involved in a system and that educational outcome retention should be measured when evaluating VR training. High-cost was the most important prohibitive barrier to VR uptake with easy access to content and low maintenance effort being most attractive factors. This study represents a first step toward international consensus around how to describe VR technology, how to approach VR educational evaluation including selection of appropriate research outcome measures, the most important barriers to technology adoption and design considerations that facilitate technology adoption into health care education.

## Introduction

Extended reality technology (XRT) is gaining interest within health care, particularly in the context of medical education, employee health and well-being, and as pain relief during invasive procedures.^1,2^ Virtual reality (VR) is a subtype of XRT and, following linguistic review of the health care literature, has been deemed complex to describe.^3^ The cited linguistic review forms part of a larger body of work interested in widening access to health care education using VR, within which, this study also sits.^3^ Throughout the health care literature, myriad contrasting descriptions for this emerging technology exists. There is no consensus around which describes it most accurately, leading to confusion within the educational and academic community.^4^ This confusion can have several negative impacts across academia, health care, and the technological ecosystem including slowing technology adoption due to lack of understanding, failure to communicate the value proposition of VR training stifling implementation, and the slowing of academic progress due to a misaligned literature base.^3^ There is a steadily strengthening evidence-base for the role of VR training in areas of emergency medicine, human factors, nontechnical skills, community-based learning, and surgery.^1,5–7^ There are examples of VR being used to replace long-standing, nationally accepted tracheostomy safety courses.^8,9^ In these platforms, equivalent educational outcomes have been obtained alongside demonstrating the opportunity to reduce the financial and environmental burden of traditional simulation.^8,9^ VR is a well-regarded tool in the armamentarium of the surgical educator involved in training laparoscopic skills, with educational benefit clear across several modalities.^10^ Despite the steadily strengthening body of data, there is no evidence or consensus guideline for the optimal way to evaluate these novel learning resources. There is a rising interest in VR in the context of medical education and as further technology developments are presented, the data presented in the literature are becoming increasingly heterogenous.^1,3^ If these teaching tools show educational and cost-effectiveness, owing to the complex nature of technology diffusion, implementation, and widespread adoption cannot be assumed.^1^

Without optimizing the environment in which XRT is expected to be embedded, uptake may be limited, thus decreasing the potential benefits within health care education. This study aimed to approach consensus agreement around terminology used to describe VR in the context of health care training, and determine the most appropriate methodological approach for its evaluation. Additionally, by identifying the most potentially prohibitive barriers to VR education adoption, we aimed to reach consensus around design and development decisions which may promote uptake within postgraduate health care systems. Ultimately, this the first step toward guideline development for researchers, educators, and clinicians interested in using, developing, and conducting research in VR education.

## Material and Methods

### Study design and ethical review

This study was an international, Modified Delphi Consensus study, which ran over two rounds in a fully-remote, online format. The study advisory group (SAG) was formed from educationalists, consultant clinicians, and medical VR experts based in several academic and care-providing institutions across the United Kingdom and the United States. Full prospective ethical approval was granted by The University of Manchester Research Ethics Committee (ref. 2023-17884-30743).

The research questions were:

1. How should we best describe VR technology in the context of postgraduate health care training?
2. What evaluation methodological practices are best used to evaluate VR training packages?
3. By what outcomes should the efficacy of VR education be best measured?
4. What are the barriers and facilitating design and development decisions that may affect the adoption and integration of VR education into medical curricula?

### Study setting and population

The study was hosted entirely online in order to balance the anticipated geographical locations of the participants, limited availability funding to support travel, and the environmental impact of running it in-person. Each round of the Delphi process was distributed using the Qualtrics^TM^ CoreXM academic survey software.^11^ Participants included senior clinicians, senior health care educators, medical students, and VR industry representatives. Participation across these four groups ensured that, broadly speaking, representation was present across a large proportion of stakeholders involved in the development, evaluation, and implementation of novel VR educational tools. For participation, an individual was required declare that they had knowledge of VR technology, although we did not assess this specifically. The study was not limited to VR experts as technology implementation is dependent on individuals far beyond the direct development and deployment such a procurement officers, educational leadership, and information technology workers. The decision was made to mandate VR knowledge as the technology is in its infancy, and nuanced, having no knowledge or understanding would significantly hamper, and potentially invalidate consensus opinions around VR educational design and implementation. Further detail of the inclusion and exclusion criteria are presented in Table 1.

**Table 1.** Detailed Inclusion and Exclusion Criteria for Study Participation

	Inclusion	Exclusion
Clinicians	Consultant level doctor in any specialty (or equivalent)	Any clinician junior to the level of consultant (or equivalent) No experience with VR technology
Educators	Senior educational role-roles may include DME, Associate DME, Dean, Associate Dean, Head of Medical School year or higher, or any equivalent educational role OR Experience of simulation design or academic evaluation OR Senior faculty on widely-established educational simulation courses such as ATLS, CCrISP, BLS, APLS, or equivalent	Insufficient educational experience such as only having experience with informal bedside teaching No experience with VR technology
Medical Students	Clinical experience on placement within a healthcare providing institution i.e., not having just experienced pre-clinical lecture periods of their training	No clinical experience No experience with VR technology
VR industry representatives	Industry representatives involved in medical VR simulation development. These may include (but not limited to) software platforms, providers, or software as a service companies, alongside hardware manufacturers or distributors Active within the last three years Hardware or software development	Experience related to activity older than three years No experience with VR technology

VR, virtual reality; DME, director of medical education; ATLS, Advanced Trauma Life Support; BLS, Basic Life Support; CCrISP, Care of the Critically Ill Surgical Patient; APLS, Advanced Paediatric Life Support.

Due to the paucity of research in the field of VR and medical education, and due to the method’s ability to provide participants with equal voice in scoring and prioritization, Delphi Survey methodology was used.^12,13^ Surveys were administered asynchronously and anonymously, with answers awarded equal attributable weight in the final scoring. Each round was self-contained, with round two running after round one had fully concluded.

Potential participants were identified and recruited using the following methods:

1. **Advertising through professional groups**—including the National Tracheostomy Safety Project, American Medical Extended Reality Association, Digital Readiness Groups at University Teaching Hospitals, and the VR@Manchester group.
2. **Purposive recruitment**—through the international networks of the research team.
3. **Snowball sampling**—utilization of the initial wave of participants by asking recruits to recommend further individuals to approach to take part in the study.^14^

Potential participants were emailed brief research descriptions and directed to the Qualtrics^TM^ CoreXM survey website where the participant information sheet was made available, and consent was received electronically before the first survey round was then immediately presented. The literature has not yet defined an optimal sample size for a Delphi Consensus study.^13^ We anticipated a minimum of 40 participants with an attrition rate of 20%–30% which is considered reasonable.^15,16^

### Analysis of Delphi rounds

Each participant was asked to score all survey points as per the Grading of Recommendations Assessment, Development and Evaluation Working Group.^17,18^ This advocated the use of an analogue visual 9-point Likert scale where 1–3 is unimportant, 4–6 is of importance but not critical to a decision, and 7–9 is of vital importance. Alternatively, where there was a binary outcome, the participants were required to answer ‘true’ or ‘false’. Definitions of consensus outlined for all question types were decided *a priori* based on Delphi methodology literature during the study design process and are outlined in detail later.^12,15,16,19^ The consensus rates for inclusion were decided with several factors in mind. Rates for inclusion are highly variable within the literature (51%–80%).^15^ The decisions outlined below balance the specific study design elements such as the wide experiences within the expert group, the timelines for data collection, and the wide variation in proposition groups and individual elements.

#### Question type: Rating of element importance

An example statement when looking at barriers to implementation of VR is as follows: “the costs of recharging the batteries of VR headsets” or “the weight of the device on the users head,” or “the risk of experiencing motion sickness.”

1. **Consensus reached for inclusion**—70% or more scored 7–9 (critically important) AND <15% scored unimportant.
2. **Consensus reached for exclusion**—less than 50% scored 7–9 (critically important).
3. **Consensus not reached**—any other combination.

#### Question type: Binary outcome statements (agree or disagree)

An example of such statement is as follows: “A VR experience should be defined by its interplay between hardware and software”:

1. **Consensus reached for inclusion**—75% agreement threshold.
2. **Consensus reached for exclusion**—<30% agreement threshold.
3. **Consensus not reached**—agreement threshold between 30% and 75%.

### Survey design and Delphi round administration

#### Proposition generation

**FIG. 1.**
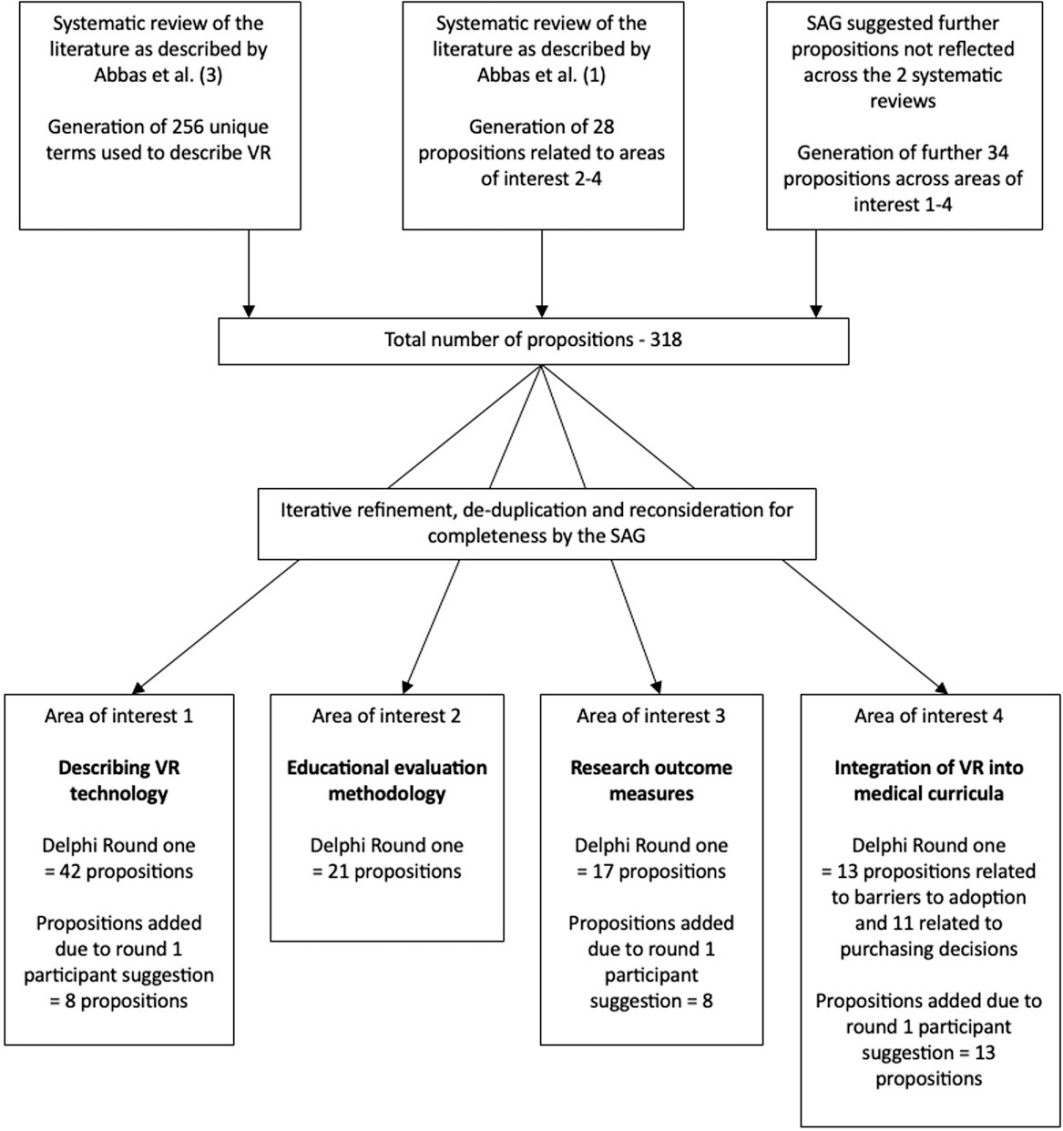
Study flow diagram depicting the generation of propositions through to study rounds one and two.

An iterative process was used to select propositions for inclusion in the Delphi process, based around two systematic reviews, published in advance, by the research team.^1,3^ From these publications, the selected propositions were reviewed by members of the SAG (I.B., J.A., E.G., and R.I.) in order to rationalize (removal of irrelevant, and removal or grouping similar propositions) and add to these for completeness and coherence. The study flow is depicted in Figure 1. The full list of presented propositions, and whether these reached consensus for inclusion or exclusion or failed to reach consensus, can be reviewed in Supplementary Data S1 and are color coded—green for consensus to include, red for consensus to exclude, and orange for propositions that did not reach consensus With the rising interest in extended reality technology, the literature base is rapidly growing. With this growth, confusion within the descriptive terminology and incoherence in the methodological approach to training tool evaluation exist. Additionally, the development expectations of the health care worker, educator, and academic are not clear. This study aimed to work toward consensus agreement around four key areas of interest: (1) descriptive terminology (2) evaluation methodology (3) research outcome measure selection, and (4) barriers and facilitators to adoption of VR into health care education. Four key areas of interest underpinned the included propositions, which were grouped as such:

**Area of interest 1—Describing VR technology (50 propositions):** Participants were presented with a list of possible definition terms that may be used to describe VR.

**Area of interest 2—Educational evaluation methodology (21 propositions):** Participants were asked to rate methodological approaches to evaluation in the context of VR training.

**Area of interest 3—Research outcome measures (25 propositions):** Participants were asked to rate outcome measures that may be used in research methodology used to evaluate VR simulation skills training.

**Area of interest 4—Barriers and facilitating design and development decisions that may affect the adoption and integration of VR education into medical curricula (37 propositions):** Participants were asked to rate barriers to integration of VR into curricula in terms of most likely to have a detrimental impact on adoption. They were also presented with propositions related to design and development principals, and asked to rate them based on likelihood to promote technology adoption.

### Two-Round Delphi process

#### Round one

All participants were asked to rate all statements across all of the areas of interest described earlier. In round one of the Delphi process, there were free-text areas for participants to suggest further propositions to be included in round.^19^ The SAG reviewed these and where appropriate included them in round two. A further free-text question was included at the end of the survey to facilitate snowball recruitment which asked the participant for recommendations for individuals they would recommend to be invited into this study.^14^ The first study round was left open for 8 weeks, a timescale which ensured there was enough time to approach further potential participants via snowball recruitment.

#### Round two

The responses to the round one survey were analyzed utilizing descriptive statistics and presented to the round two participants. Additionally, participants in round two were shown their own answers from round one. Areas which reached consensus (described later) were not re-questioned in round two of the survey in order to improve efficiency when using participants time and improve the attrition rate.^16^ Round two was left open for 4 weeks and written reminders were sent via email to all participants who participated in round one.

## Results

Forty-one participants completed the first Delphi round and 26 completed the second. Of the 41 total first round participants, 36 were based in Europe (including outside the United Kingdom) and five in North America. In the second round 24 participants were based in Europe and two in North America. Participants self-reported their single, or multiple professional roles to be senior educators (round one *n* = 23, round two *n* = 15), senior clinicians (round one *n* = 16, round two *n* = 14), VR industry experts (round one *n* = 5, round two *n* = 4), or undergraduate medical students (round one *n* = 3, round two *n* = 0). Experience with VR was varied, with all participants in round one having knowledge of the technology, and 20 participants actively contributing or leading design of VR educational packages. Full details of all participants are summarized in Table 2. Across all areas of interest delineated later, the group were able to reach consensus for inclusion for 49 individual propositions by the end of round two.

**Table 2.** A Table Summarizing the Personal Details of the Participants Recruited to the Study Including Professional Role, Location of Work, and Level of Experience with VR Technology

	Participant factor	Number (%)
Professional Role	VR industry professional	5 (10.6)
	Senior doctor	16 (34.0)
	Senior medical educator	23 (48.9)
	Medical student	3 (6.4)
Location of Work	Europe	36 (87.8)
	United States of America	5 (12.2)
Experience with VR technology	Knowledge of VR	6 (14.6)
	Used VR but not directly involved in design of educational interventions	15 (36.6)
	Contribute but not lead the design of VR educational interventions	6 (14.6)
	Lead the design of VR educational interventions	14 (34.2)

VR, virtual reality.

### Area of interest 1—describing VR technology (first research question)

A total of 42 key terms were initially presented to the participants in round one. During this round, an additional eight terms were suggested to be reviewed in round two by the participants. Of the 50 total terms presented to the participants across both Delphi rounds, 15 reached the consensus level for inclusion and therefore deemed highly relevant when describing VR technology. Terms which reached the highest level of agreement for use when describing VR technology included: ‘virtual environment’, ‘interactive’, and ‘simulation’. Additionally, 38 (95.0%) of the participants agreed that “A VR experience should be defined by its interplay between hardware and software.” A full breakdown of all of the propositions included by consensus across all areas of interest are displayed in Table 3.

**Table 3.** A Summary of All Propositions throughout the Four Key Areas of Interest Which Reached the Level of Consensus for Inclusion Across Both Delphi Rounds

	No.	Proposition (see Supplementary Data S1 for full description of each proposed term or statement)	Agreement (%)
Describing VR technology	1	Virtual Environment (a digital simulated space which can be real or fictional).	95.1
	2	Interactive (e.g., buttons, choices, or fully-interactive elements).	95.0
	3	Simulation (imitation of an environment, object or process).	90.2
	4	Environment (e.g., an operating theatre, emergency room, or fictional area).	84.6
	5	Immersion (relating to the sensations that a user experiences).	82.9
	6	Interactive Simulation (an imitated environment, object, or process which can be interacted with).	82.9
	7	3D (digital assets or environments with the three spatial dimensions of height, width, and depth).	80.8
	8	Experience (an activity or event that the user experiences which may be passive or active).	80.5
	9	Technology (including hardware, software, or both).	80.5
	10	3D Environment (combination of the above-described terms ‘3D’ and ‘environment’).	76.9
	11	Display (an electronic screen-based component from which a user experiences digital media such as a screen or projection).	75.6
	12	Reality (an experience which is representative of real-life).	73.2
	13	Head Tracking (software which is aware and responds to the position of a user’s head).	73.2
	14	Human Computer Interface (wide-ranging technological term describing the point where a user interacts with a computer-based device).	72.0
	15	Head Mounted Display (a type of VR device worn on a user’s head which displays media via screens to each, or both eyes).	70.7
Educational evaluation methodology	16	Outcomes should be measured again after a period to assess retention.	89.5
	17	Mixed methods research (qualitative and quantitative data) is the best approach when evaluating VR education. Example seen in a study by Ryan et al.^20^	84.2
	18	VR educational modalities should be focused around Kirkpatrick learning level 3 as described as the transfer of knowledge, skills and abilities.	80.0
	19	Usability should be measure by validated questionnaires such as the widely accepted System Usability Scale.^21^	76.0
	20	Content validity is mandatory for the validation of VR training tools. An example may be seen in a recent study by Henniger D at al.^22^	75.7
Research outcome measures	21	Knowledge gained from using the VR simulation. Knowledge can be theoretical or practical and usually measured using multiple-choice questions.	100.0
	22	Overall system usability. Measure of how easily a user can use a system for a specific goal.	94.7
	23	Skill performance in real life situation at least 3 months after training. Long term skill retention indicates longevity of educational outcomes.	92.1
	24	Organisation outcomes such as adherence to standards, grouped patient outcomes, or litigation spend.	92.1
	25	Skill performance in subsequent VR simulation. This represents how well a user performs the taught clinical skill measured within a VR simulation.	89.5
	26	Skill performance in real life situation soon after training. This represents how well a user performs the taught clinical skill measured in a real-life situation.	89.5
	27	Skill performance in subsequent simulation. This represents how well a user performs the taught clinical skill measured in simulation.	86.8
	28	User experience-satisfaction. Was the user happy with the experience?	86.8
	29	Reporting of adverse effects such as nausea, disorientation, or headaches which would indicate low safety or low levels of satisfaction as a result.	84.2
	30	User experience-level of engagement.	84.2
	31	User perceived educational effectiveness. Measuring from the users’ perspective whether they learnt anything from the experience.	82.1
	32	Patient outcomes following administration of VR training. Measure of whether a VR experience has impacted patient outcomes such a pain, bleeding, length of stay etc.	81.6
	33	User experience-level of motivation.	80.0
	34	Analysis of ease of deployment. Building on usability but focussed on setup and running of the VR system.	80.0
	35	Cognitive workload. An example measure is the Nasa Task Load Index.^23^ This is the mental effort required to complete a task.	76.3
	36	User experience-realism.	73.7
Integration of VR into medical curricula: barriers to technology adoption	37	High cost. This can limit uptake of new technologies due to budget constraints by decision makers.	78.9
	38	Lack of evidence of educational benefit. Evidence may be in the context of research or informal evaluation. Low evidence may affect educational decision makers to adopt.	76.0
	39	Digital readiness. If a healthcare institution is not ready to adopt new digital technologies, then uptake cannot take place.	76.0
	40	Lack of clarity of how a simulator is used within a curriculum. A clear education or training use-case is required.	75.0
	41	Patient specific factors not taught. Specific factors may include anatomy ahead of a specific intervention, or considering particular individual patient factors.	72.0
Integration of VR into medical curricula: Design decisions	42	Easy access for your trainees or students to use the system. This may be measured assessing the usability of the system. Additionally features such as not requiring internet connectivity, or device agnosticism may be improve access.	97.4
	43	Low maintenance cost. Maintenance costs may be incurred in the exchanging of batteries, charging headsets or refreshing degradable consumables that are required in some VR experiences.	94.7
	44	High level of graphical fidelity i.e., it looks more realistic.	91.7
	45	The ability for your students to tolerate the experience for longer periods of time. Meaning that the side-effect profile is low enough to ensure comfort for longer.	87.5
	46	Large volume of content or frequent updates with more content. This may be in the form of multiple experiences, longer experiences, or sufficient depth to promote repeat use.	79.2
	47	Low chance of adverse effects (e.g., sickness, disorientation). Abbas et al. reported the majority of VR systems had no side effects in a systematic review of VR emergency simulations.^1^	79.2
	48	Low up-front cost of purchasing the system. This is a relative factor to the individual circumstances and funding level of a particular institution.	76.3
	49	Low chance of technical issues. These may take many forms but commonly may include connectivity or reliability issues.	76.3

VR, virtual reality.

Several terms did not reach consensus after the two round Delphi process including ‘engaging’ (65.4%), ‘active’ (65.4%), ‘virtual world (65.4%)’, ‘system’ (57.7%), ‘multi-sensory’ (57.7%), ‘3D image’ (57.7%), ‘natural hand motion/tracking’ (50.0%), ‘activity’ (42.3%), and ‘high fidelity’ (50.0%).

### Area of interest 2—educational evaluation methodology (second research question)

In order to explore their opinions about the most appropriate methodology when evaluating novel VR educational tools, 21 statements were presented to the participants over both Delphi rounds. Of these, a total of five statements reached the level for consensus agreement. Over 80% of participants agreed that mixed methods should be utilized when evaluating educational tools and studies that should measure educational outcomes over multiple time periods in order to determine retention of learning (propositions 16 and 17 in Table 3, respectively).

Ten out of the presented 21 statements did not reach consensus for over the two-round Delphi process. These are included, alongside their levels of agreement in Table 4.

**Table 4.** A Table Outlining the Propositions within the Educational Evaluation Methodology Area of Interest and Associated Percentage Rate of Agreement after the Delphi Process

Proposition (see Supplementary Data S1 for full description of each proposed term or statement)	Agreement (%)
Face validity is mandatory for the validation of VR training tools	72.0
As long as conflicts of interest are declared, evaluations can be performed by the intellectual property owner of the VR software	64.0
Novel VR educational modalities should be focused around Kirkpatrick learning LEVEL 2	64.0
Evaluations can be single-centre	64.0
Criterion validity is mandatory for the validation of VR training tools	60.0
When measuring skill transfer following VR training, this must be using validated rating scales such as the Global Rating Scale	56.0
Multiple centres within the same country are required for successful validation of VR education	52.0
Novel VR educational modalities should be focused around Kirkpatrick learning LEVEL 4	40.0
Evaluations can be observational in nature and therefore not require a comparator	39.1
Construct validity is mandatory for the validation of VR training tools	36.0

VR, virtual reality.

### Area of interest 3—research outcome measures (third research question)

A total of 17 potential research outcome measures were initially presented to the participants in round one. During this round, an additional eight outcomes were suggested by the participants in the white space area to be included in review during round two. A total of 25 outcomes were therefore assessed and 16 reached the consensus level for inclusion. The participants unanimously agreed that the participant’s knowledge should be measured when evaluating the learning content of a VR training tool. In addition, usability, skill performance retention, and organizational outcomes reached the level for consensus inclusion with rates of agreement over 90%.

Five of the proposed outcome measures did not reach consensus opinion including ‘stress measured with user reported questionnaires’ (68%), ‘net promotor score’ (52.0%), ‘cost analysis’ (56.0%), ‘measure of system longevity’ (64.0%), and ‘assessment of team working’ (56.0%).

### Area of interest 4—integration of VR into medical curricula (fourth research question)

Thirteen individual barriers to adoption of VR technology were presented to the participants in round one where an additional 13 barriers were suggested for assessment. Of the 26 presented barriers, consensus agreement for inclusion was achieved identifying the six most prohibitive barriers to adoption of VR training. High cost achieved the highest level of agreement (78.9%).

When asked to rate 11 additional statements relating to priorities when deciding to procure and implement VR training tools seven statements reached consensus for agreement and therefore deemed the most desirable characteristics of a VR system. Easy access for students, low maintenance cost, and high graphical fidelity were priority areas reaching consensus with an agreement level of over 90% (propositions 42–44 in Table 3).

Of the propositions presented related to barriers of adoption of VR technology six did not reach the level of consensus opinion including ‘low simulator fidelity’ (62.5%), ‘lack of assessment, feedback or progress monitoring’ (68.0%), ‘skills of facilitator/educator’ (64.0%), ‘learning curve required to overcome’ (66.7%), ‘time constraint to implement’ (56.0%), and ‘resistance to change’ (68.0%). Additionally, when asked about development priorities, consensus agreement was not reached when asked to consider the importance of ‘generating content yourself’ (66.7%).

## Discussion

This study represents a first step toward the development of an expert, consensus-based, multidisciplinary guideline describing the description, development, and validation of VR technology in the context of health care training.

Consensus is difficult to achieve, and it is well-described in the literature that language around VR and other immersive technologies is confusing, lacks consistency, and may lead to a detrimental effect on innovation within the health care educational system.^3,24^ Reasons for such complexity include a long history of technology development, branching and combining of different technology subtypes, and rapidly growing research and educational interest without interdisciplinary agreement around definitions.^3,25^ Often technology descriptions become obsolete due to hardware advances, but continue to be referenced in the academic evidence base. As presented in Table 3 several terms were highly relevant to the consensus group. Terms such as ‘head mounted display’ (HMD) have recently become more commonplace in the literature, and have been specifically identified as important.^3^ Across the XRT industry, not all VR experiences are delivered via HMDs, but the expert consensus group found it to be an important descriptive feature, if relevant, for the purpose of health care applications and research. Whilst some hardware-related terms were considered important by consensus, the majority of terms related to the experience that a user would have. Consensus agreement that VR experiences are interactive, immersive, and 3D, attest to the fidelity that VR technology can provide over other traditional more established screen-based technologies.

Interestingly, many of the terms which commonly occur in the literature have reached consensus for inclusion when defining VR by this consensus group including: ‘environment’ ‘interactive’, ‘simulation’, ‘virtual environment’, ‘3D’, ‘experience’ and ‘technology’, and ‘head mounted display’.^3^ The term ‘computer’ did not reach consensus despite being the second most commonly occurring term in medical literature definitions of VR.^3^ It is a broad term, and avoiding the use of it may add clarity to VR descriptions. The 15 terms reaching the level of consensus for inclusion (summarized in Table 3), provide some guidance to clinicians, educators, and institutions when describing VR in their work. By using these terms where relevant, it is more likely that the technology they wish to describe is understood by their intended audience. In addition to recommendations on individual descriptive terms used to describe VR, the Delphi participants almost unanimously agreed that any literature description of novel health care VR should be described using the hardware and software interplay, and not by hardware, or software alone. This point should at least act as a general approach to describing VR technology if the specific terms are not used. It is a simple principal, likely to communicate descriptions of VR in an understandable way.

VR simulation skills training is gaining interest throughout a wide range of health care disciplines.^1,26–29^ There is a growing literature-base around evaluation of educational VR, but consensus guidelines informing evaluation do not exist. The National Institute of Healthcare Excellence have published guidance on evaluation of health care technology, but is limited to therapeutic, diagnostic, and medicinal applications which require Medicines and Healthcare products Regulatory Agency approval, which VR education does not.^30^ Guidelines such as these enable innovators to navigate the complex and stringent evaluation requirements of medical technologies.^30^ Without consistent, high-quality data, one cannot combine study findings and make evidence-based recommendations that go beyond a single pilot implementation of VR. The Delphi group agreed on five general evaluation approaches (propositions 16–20 in Table 3). There was agreement that content validity was mandatory in the context of novel educational VR tool development and describes the “degree to which the content of a simulator precisely portrays the intended medical construct”.^31^ There are many ways to measure this, and using expert ratings of usefulness and satisfaction may offer a cost-effective, viable option which satisfied the Delphi consensus opinion that content validity assessment should be performed. It is a process, already well-established in the simulation education, and technology enhanced learning literature, albeit with the accepted limitations of subjectivity of raters, and the issues that arise when the content domains are not well defined.^32–35^ Further to this, although not quite meeting the threshold for consensus, face-validity was an approach that the majority of our participants deemed important.

The expert group recommend a mixed-methods approach when evaluating novel VR training. In contrast to traditional interventional research, randomized controlled trial (RCT) study design approaches were not recommended. Whilst RCTs are considered the gold standard for effectiveness studies, within the field of medical education, limitations include high cost, low potential generalizability of the results and the lead time associated with the running of this type of study often prohibit their use.^36–38^ Similarly, multi-site studies were not deemed mandatory, a viewpoint which pragmatically limits scaling research costs. High resource requirements have a compounded effect when evaluating technology enhanced learning tools. Prompt, often cost-effective evaluations are required, due to the speed at which the technology is advancing and that prototype simulations are often built in a low budget, often grant funded environment.^39^

The Topol review, suggests the future importance of XRT in education and patient care alongside several other technologies such as genomics and artificial intelligence.^40^ On review of the literature to-date, there have been no positive steps toward development of guidelines supporting educational innovation using XRT, as there have been for medical devices.^30^ This study is a first step in the development of such guidelines. If we are to see significant level of uptake, it is of vital importance that the presented barriers to technology adoption are considered. Addressing these are the responsibility of the product owners, associated educational researchers, device manufacturers and the institutions that they are being implemented within.^41^

Finally, technology adoption within any health care system is reliant on sustainable business models.^42^ Our Delphi group have agreed on several design and development decisions which should be considered as these are likely to steer purchasing decisions, driving revenue, and promoting a viable business behind the development of technology. These priorities are centered on three main areas including cost, volume of educational content, accessibility, and comfort (propositions 42–49 in Table 3). In terms of cost, we have reported in prior work the lack of evidence reporting cost of development and integration of VR.^1^ This, in turn, risks not providing interested institutions with the information required to explore this area further.^1^ Concerns about cost are not limited to VR and technology enhanced learning, but traditional simulation skills training also.^43^

## Strengths and Limitations

Delphi studies have inherent strengths when compared to other methods of qualitative research.^44^ Providing the participants in the expert consensus group an opportunity to revise their choices based on the choices of the whole group offers an opportunity for critical reflection, not possible in single focus group, interviews, or questionnaire exercises.^44^ Due to the asynchronous approach the anonymity supported honest feedback, providing equal opportunity regardless of personality dominance, or professional seniority which could affect the responses of others. A further strength of this study is that it gathers consensus opinion from a wide range of stakeholders from different backgrounds, with knowledge of VR technology.

The inherent weaknesses of the Delphi method is the high dropout rate due to the time consuming nature and complexity of a study with multiple rounds. We observed a 63.4% two-round completion rate which was lower than anticipated, which may be criticized when considering the overall validity of the study findings. The introduction of nonresponse bias and self-selection bias are two of the key impacts of a high dropout rate.^45,46^ This may skew the perspectives toward a population which are more likely to take part fully in a study such as this. Other design choices may be questioned such as the decision to focus on the individuals or institutional representatives involved in the design and implementation of VR in education, rather than the end-user/learner. This would be a valid area of focus if the scope of this study is to be widened.

## Future Directions and Practical Applications

This study is the first attempt at exploring consensus agreement around the development and implementation of VR in the context of health care education. The next step for this work is to formally collaborate with representatives from the stakeholder groups included here together with relevant educational and regulatory organizations to develop guidelines to support the design, implementation, and evaluation of novel VR training tools. Consensus guideline development in this area will support stakeholders as uptake of the technology accelerates, enabling more efficient progress across all stages of development and implementation. As part of this process, further work is required to on the foundation that this study contributes. In each of the areas of interest described above, several propositions failed to reach consensus agreement for inclusion or exclusion. It wouldn’t be expected to reach consensus in every area, however well-constructed qualitative research exploring the more potentially high-impact areas would be beneficial as we move toward guideline development. Many propositions did not reach consensus around most appropriate evaluation research methodology, representing a clear area for focus. Cost was identified as one of the most significant barriers to adoption of this technology, yet our multidisciplinary group didn’t agree that cost analysis was a sufficiently important outcome measure for inclusion. Considering this further exploration of this area through focus groups and structured interviews of appropriate stakeholders would be beneficial. Importantly, these guidelines will include information to support the description of VR within the literature and across education, technology, and academic institutions to ensure effective communication, in an attempt to ensure confusion is kept to a minimum, especially as the XR ecosystem matures.

Ahead of comprehensive consensus guideline development, there are several practical applications of the study findings. Our study recommends the use of several descriptive terms for use when communicating VR educational technology. These can be directly used in describing novel applications, reporting research, and in the development of courses or curricula using VR technology. Understanding and experience of the technology amongst health care workers is limited, and supporting streamlining of the descriptive terminology is key to simplifying the ecosystem.^25^ Additionally, these results help guide researchers planning educational evaluation, and educational institutions when deciding how to develop or procure XRT enhanced educational tools.

## Conclusion

This study clearly outlines the views of a multidisciplinary group around several areas of interest. We explore how the expert group reached consensus around how to describe VR technology in the context of health care education, approach evaluating novel VR training tools, select appropriate research outcome measures, and most important barriers to technology adoption alongside design and development principles to promote adoption. Design of technology enhanced learning relies on a balance of experience and the limitations of both resource and time. By understanding the priorities of the multidisciplinary stakeholder group, this work can inform design choices, evaluation study design, and work toward a more consistent body of language around a rapidly evolving, potentially highly beneficial space. We can move toward guidelines to support the evaluation of novel VR technology enhanced tools invaluable to the developers, educators, clinicians and ultimately learners who are expected to use these tools to learn.
